# Antimicrobial and anti-biofilm properties of oleuropein against *Escherichia coli* and fluconazole-resistant isolates of *Candida albicans* and *Candida glabrata*

**DOI:** 10.1186/s12866-024-03305-5

**Published:** 2024-05-04

**Authors:** Mohammad Ali Esfandiary, Ali Reza Khosravi, Sepideh Asadi, Donya Nikaein, Jalal Hassan, Aghil Sharifzadeh

**Affiliations:** 1 Department of Microbiology and Immunology, Faculty of Veterinary Medicine, University of Tehran, PO Box: 14155-6453, Tehran, Iran; 2 Department of Comparative Biosciences, Faculty of Veterinary Medicine, University of Tehran, Tehran, Iran

**Keywords:** Anti-biofilm, Oleuropein, *Escherichia coli*, *Candida albicans*, *Candida glabrata*

## Abstract

**Background:**

Side effects associated with antimicrobial drugs, as well as their high cost, have prompted a search for low-cost herbal medicinal substances with fewer side effects. These substances can be used as supplements to medicine or to strengthen their effects. The current study investigated the effect of oleuropein on the inhibition of fungal and bacterial biofilm in-vitro and at the molecular level.

**Materials and methods:**

In this experimental study, antimicrobial properties were evaluated using microbroth dilution method. The effect of oleuropein on the formation and eradication of biofilm was assessed on 96-well flat bottom microtiter plates and their effects were observed through scanning electron microscopy (SEM). Its effect on key genes (*Hwp1, Als3, Epa1, Epa6, LuxS*, *Pfs*) involved in biofilm formation was investigated using the quantitative reverse transcriptase-polymerase chain reaction (RT-qPCR) method.

**Results:**

The minimum inhibitory concentration (MIC) and minimum fungicidal/bactericidal concentration (MFC/MBC) for oleuropein were found to be 65 mg/ml and 130 mg/ml, respectively. Oleuropein significantly inhibited biofilm formation at MIC/2 (32.5 mg/ml), MIC/4 (16.25 mg/ml), MIC/8 (8.125 mg/ml) and MIC/16 (4.062 mg/ml) (*p* < 0.0001). The anti-biofilm effect of oleuropein was confirmed by SEM. RT-qPCR indicated significant down regulation of expression genes involved in biofilm formation in *Candida albicans* (*Hwp1, Als3*) and *Candida glabrata* (*Epa1, Epa6*) as well as *Escherichia coli* (*LuxS, Pfs*) genes after culture with a MIC/2 of oleuropein (*p* < 0.0001).

**Conclusions:**

The results indicate that oleuropein has antifungal and antibacterial properties that enable it to inhibit or destroy the formation of fungal and bacterial biofilm.

## Background

Biofilms are complex structures of living microorganisms that they form through the creation of a matrix of extracellular polymeric substances attached to living or non-living surfaces. Biofilm protects microorganisms from environmental factors such as antibiotics and other aspects of the immune system and is considered to be a virulence factor [1]. Generally, drug sensitivity tests for antifungal agents assessing the inhibition rate of microbial species are conducted on planktonic microbial populations and have not been used for biofilm forms [2]. Therefore, this has made it very difficult to establish a relationship between antifungal agent sensitivity results and their effects on microbial biofilm forms [3, 4]. An increase in biofilm antifungal agent resistance is caused by the higher number of cells that make up the community within the biofilm, effects of the biofilm matrix, a decrease in growth rate, restriction of access to food and/or upregulation of resistance genes, especially those of resistance pumps [5].

*Candida* spp. is among the most important cause of fungal infection in humans and animals. These infections are more common in people with underlying factors and vary from mucosal colonization to invasive and fatal infections. *Candida albicans* is a common cause of such infections. *Candida glabrata* is another common species that is found on the surface of the body and has been isolated from the skin and urine. *C. albicans* and *C. glabrata* are considered to be an opportunistic agent of superficial and visceral fungal diseases in humans [6].

One of the factors that contribute to the virulence of *C. albicans* and *C. glabrata* is their ability to form biofilm. *C. glabrata* biofilm was promoted in the presence of an increase in the amount of serum due to oral inflammation induced by denture plaque [7]. Stents, implants, endotracheal tubes and catheters are important devices that are prone to biofilm formation and can be a source of successive infections. *C. albicans* is known as the third most common cause of catheter infections, which may be related to the complications of biofilm formation of this fungal species [8].

*Escherichia coli* is a common bacterium found in the normal intestinal flora and is globally associated with disease outbreaks, such as dysentery and hemolytic uremic syndrome (HUS) [9]. Also, *E. coli* biofilm is related to various diseases, including urinary tract infections [10] and Bloodstream Infections [11]. There have been concerns about the recurrence and treatment of mentioned diseases [12, 13]. Studies have reported increasing resistance of this bacterial biofilm to antimicrobial drugs [14, 15]. *E. coli* biofilms protect against antibiotic treatment and the immune system. They can be up to 100–1000 times more resistant to antibiotics than plankton bacteria [16]. Antibiotic resistance is mainly attributed to various mechanisms including low antimicrobial penetration, reduced growth rate and stress responses, persisted cells, changes in efflux pumps [17].

Hyphal wall protein 1 (*Hwp1*) is a fungal cell wall mannoprotein that promotes adhesion of *Candida* cells to a host surface [18] and biofilm formation [19]. Studies have shown that this gene also plays a role in the formation of germ tubes and hyphae forms which promote physical contact between epithelial cells and fungi. It can be concluded that *Hwp1* is an important factor in pathogenicity and biofilm formation in *C. albicans* [20].

Adhesion in *C. albicans* is mediated by a group of agglutinin-like sequence proteins (*Als*). In particular, *Als1* and *Als3* play important roles in biofilm surface attachment [21]. The *Als* gene family consists of eight genes, of which *Als3* is the most prominent. *Als3* is involved in adhesion to host tissue as well as adhesion to bacterial species [22–24]. Adhesion by *C. glabrata* occurs through epithelial adhesives called *Epa*, which have a structure similar to *Als* proteins. The *Epa* gene family consists of 17 to 23 genes, of which *Epa1*, *Epa*6 and *Epa7* are the most important adhesives [25]. The *luxS* and *Pfs* genes are among the most important genes involved in the production of *E. coli* biofilm and can cause intestinal and wound colonization [26]. During the stages of biofilm formation of gram-negative bacteria, including *E. coli*, autoinducer 2 (AI-2) signals derived from furanones increase biofilm formation, which results in increased pathogenicity [27]. In quorum sensing (QS) pathways, *luxS* and *pfs* genes are important genes involved in AI-2 synthesis [28].

The main cause of bitterness in the fruit of olive trees is oleuropein. It is one of the most important and abundant phenolic compounds in the fruit and leaves of olive trees (*O. europaea* L.) [29]. This compound is the dominant bioactive compound in the olive leaf and can be purified using different methods [30]. Oleuropein offers antimicrobial, antioxidant, anti-inflammatory, anti-atherogenic, anti-cancer and anti-aging properties as well as skin protection [31]. Oleuropein belongs to a group of coumarin compounds known as secoiridoids and is the most abundant bio phenol in olive leaves [32]. Similar to other phenolic compounds, oleuropein shows its antimicrobial effect through the destruction of cell membranes and the peptidoglycan. Several studies have shown interactions between oleuropein and membrane lipids. The ortho-diphenol structure of oleuropein has been shown to be the active agent of its antimicrobial mechanism. However, its mechanism has not been fully elucidated yet [33] Because oleuropein is a free radical scavenger, it has very strong antimicrobial activity [34].

Over the years, medicinal plants have been utilized as substitutes for various drugs and chemical disinfectants to eradicate biofilms. The present study aimed to determine the minimum inhibitory concentration (MIC) and minimum fungicidal/bactericidal concentration (MFC/MBC) of oleuropein, a medicinal substance found in olive leaves, and to investigate the abilities of inhibiting the formation of biofilm and destroying the biofilm of *E. coli* and also fluconazole-resistant isolates of *C. albicans* and *C. glabrata*. This investigation was carried out in-vitro and at the molecular level.

## Material and methods

### Organism culture media and chemicals

*C. albicans*, *C. glabrata* and *E. coli* obtained from Mycology Reference Center and Bacteriology Collection, faculty of Veterinary Medicine, University of Tehran. The *Candida* yeast was cultured on Sabouraud dextrose agar (SDA; Merck, Germany) and the *E. coli* bacteria were cultured in Mueller–Hinton broth medium (MHB; pH: 7.2–7.4, Merck, Germany) at 37 °C for 16 to 18 h.

*Candida parapsilosis* ATCC 22019 and *Candida krusei* ATCC 6258 were used to control the validation of fluconazole susceptibility test method, according to CLSI M60 standard.

The oleuropein (purity 98%) was acquired from the Department of Comparative Biosciences, Faculty of Veterinary Medicine at the University of Tehran. The fluconazole was acquired from Sigma-Aldrich (UK; 98%; CAS number 86386–73-4).

### Antimicrobial sensitivity test on planktonic cells

The MIC/MFC of *Candida* yeast for oleuropein and fluconazole were determined using the microbroth dilution method according to CLSI M60-Ed1 document [35]. The MIC/MBC of *E. coli* for oleuropein was determined using the microbroth dilution method according to CLSI M100-Ed31 guidelines [36].

For drug susceptibility testing of *Candida* yeast, two-fold serial dilutions of 260 to 0.2 mg/ml of oleuropein and 256 to 0.5 μg/ml of fluconazole were prepared in RPMI-1640 medium (GIBCO-BRL) with 0.1% dimethyl sulfoxide (DMSO). MHB was used to test the drug sensitivity of the *E. coli* bacteria.

The final number of yeast suspensions (0.5 × 10^3^ to 2.5 × 10^3^ colony forming units (CFU)/ml) and bacterial suspensions (5 × 10^5^ CFU/ml) were prepared using a hemocytometer slide. They were then added to all wells, except for the well designated as the negative control, in each row. In each repetition of every experiment, one well was considered as a positive control in each row and specifically for each microorganism, and this well contained culture medium with suspension inoculation. The plates were slightly mixed and then were placed in a 37 °C incubator for 24 h. The lowest concentration of oleuropein in a well in which no turbidity was visually observed was considered as the MIC value. The MBC/MFC value was determined using 10 μl from wells showing no visible growth inoculated on Tryptone soy agar (TSA; Merck; Germany) for the *E. coli* bacteria and SDA (Merck, Germany) for *Candida* spp., respectively. The MBC/MFC value was defined as the minimum concentration of drug where no growth had been observed [37]. These tests were performed in triplicate.

### Effect of oleuropein on biofilm formation

Biofilm formation was determined using a modified version of a previously described quantification method [38–41]. Initially, two to four colonies that had been cultured in solid medium were grown in yeast peptone dextrose (YPD; Merck; Germany) broth medium (for *Candida* spp.) and Tryptic soy broth (TSB; Merck, Germany) medium (for *E. coli*) overnight at 37 °C. The resulting suspension was then centrifuged and the supernatant was drained off. The yeast sediment was washed twice with sterile Phosphate buffered saline (PBS). Subsequently, the number of cells in the final suspension was adjusted to 10^6^ in RPMI-1640 medium for the *Candida* spp. and TSB medium for *E. coli*, using a hemocytometer slide.

In this study, flat-bottom tissue culture microplates were used. One hundred μl sterile 96-well plates were filled with concentrations of MIC/2, MIC/4, MIC/8 and MIC/16 of oleuropein mixed with broth medium containing 0.1% DMSO. Buffered RPMI-1640 culture medium was used for the yeast, while TSB medium was used for the bacteria in accordance with the instructions for biofilm formation. An equal volume of each prepared inoculum was added to the wells of the 96-well microplate. Positive controls containing suspensions prepared from a specific organism and the negative controls without it were included.

The microplates were incubated at 37 °C for 24 h. After the incubation period, the supernatant solution was removed and the wells were gently washed three times using PBS. Then, biofilm measurement was carried out using The MTT (3-(4,5-dimethylthiazol-2-yl)-2,5-diphenyltetrazolium bromide) assay [42]. The MTT kit (DNA Biotech; Iran) was used following the manufacturer instructions. A total of 1 ml of PBS was added to 5 mg of tetrazolium salt and mixed well. This stock solution can be stored frozen if not used immediately. Next, 0.5 ml of stock solution was added to 4.5 ml of prepared PBS and 100 μl of the resulting mixture was added to each well containing biofilm. After 4 hours of incubation at 37 °C in the dark, due to the sensitivity of the reaction to light, the supernatant was discarded and was added 100 μl of DMSO. After approximately 15 min, blue formazan crystals appeared and the optical density (OD) of each well was measured using a microplate reader (ELx808; BioTek; USA) at 560 nm. The biofilm inhibition percentage was calculated using the following formula: 100 - [(OD_560nm_ of treated wells)/(mean OD_560nm_ of control wells without antimicrobial agent) × 100)] [43]. Each test was repeated three times to ensure the accuracy and consistency of the results.

### Effect of oleuropein on preformed biofilms

A total 100 μl of the prepared suspension was inoculated into the wells of a 96-well plate and incubated at 37 °C for 24 h. The supernatant was then removed and the biofilm was gently washed three times with PBS. Next, 100 μl of specific dilutions of oleuropein were added to each well to achieve a final concentration equivalent to MIC/2, MIC/4, MIC/8 and MIC/16. After 24 h of incubation at 37 °C, the contents of the wells were emptied and the wells were again washed three times with PBS. Finally, the amount of biofilm was determined using The MTT assay (see Section 2.3). This experiment was carried out in triplicate and the average values obtained were used for calculations. Biofilm destruction was determined, calculated employing the formula as described in the previous section.

### SEM of oleuropein on structural cells and biofilm

One millilitre of buffered RPMI-1640 with 0.1% DMSO containing an MIC/2 concentration of oleuropein was added to each well of the sterile 24-well plates, along with 1 ml of prepared microbial inoculum. In the next well, a mixture of culture medium and yeast or bacteria suspension was used instead of the oleuropein solution. Also, a suspension of *C. albicans* and *E. coli* (1: 1 ratio) was used to observe the effect of oleuropein in the biofilm of the microbial mixture according to the above method. The biofilm formed on very thin PVC slides measuring 7 mm which were placed inside the wells and incubated for 48 h at 37 °C.

After incubation, each slide was removed and washed with PBS, followed by fixation with 2.5% glutaraldehyde for 2 h at 4 °C. The samples were then dehydrated using alcohol concentrations of 30, 70, 80, 90, 95 and 99%. After coating the samples with a layer of gold, the three-dimensional structure of the samples was imaged using a JEOL JSM-840 scanning electron microscope [41, 44].

### Effect of oleuropein on gene expression

The RT- qPCR method was used to investigate the inhibitory effect of oleuropein on gene expression in the biofilm of *C. albicans* (*Hwp1, Als3*), *C. glabrata* (*Epa1, Epa6*) and *E. coli* (*LuxS*, *Pfs*). The primers were designed based on sequences that have been reported in previous studies (Table 1). RNA extraction and cDNA synthesis were performed using the method described in the next section.

**Table 1:** Forward (FW) and reverse (RV) primers used in study [45, 46]

Primer sequence 5′ to 3′	Gene
TGCTGAACGTATGCAAAAGG	*ACT1_alb FW*^a^
TGAACAATGGATGGACCAGA	*ACT1_alb RV*^a^
TTGCCACACGCTATTTTGAG	*ACT1_gla FW*^a^
ACCATCTGGCAATTCGTAGG	*ACT1_gla RV*^a^
TCTACTGCTCCAGCCACTGA	*Hwp1 FW*
CCAGCAGGAATTGTTTCCAT	*Hwp1 RV*
CTGGACCACCAGGAAACACT	*Als3 FW*
GGTGGAGCGGTGACAGTAGT	*Als3 RV*
ATGTGGCTCTGGGTTTTACG	*Epa1 FW*
TGGTCCGTATGGGCTAGGTA	*Epa1 RV*
TTATGCCGTATGGGGTTCTC	*Epa6 FW*
GAGTCAACTGAGGCACACGA	*Epa6 RV*
ATACCGCATAACGTCGCAAG	*rrsD FW*^a^
ATATTCCCCACTGCTGCCTC	*rrsD RV *^a^
AATCACCGTGTTCGATCTGC	*LuxS FW*
GCTCATCTGGCGTACCAATC	*luxS RV*
ATCGTTGTCTCGGACGAAGC	*Pfs FW*
GGACAGCCTGGTAACTGACCG	*Pfs RV*

^a^*The housekeeping genes*

### RNA extraction and cDNA synthesis

The biofilm was prepared in accordance with Section 2.3, with and without a MIC/2 concentration of oleuropein. The well without treatment with oleuropein is considered as the positive control and the well containing only the culture medium as the negative control for each microorganism and in each repetition. After 48 h of incubation at 37 °C, the biofilm was washed with PBS and RNA extraction was performed using an RNA Extraction and Purification Kit (Jena Bioscience; Germany) according to the manufacturer protocol. The cDNA then was obtained using a commercial cDNA synthesis kit (Sina Clon; Iran).

### Reverse transcription quantitative PCR

Real-time PCR was performed using the SYBR Green master mix kit (Ampliqon; Denmark) and primers for housekeeping genes (*ACT1-alb, ACT1-gla, rrsD-Ecoli*) and putative virulence genes (*Hwp1, Als3, Epa1, Epa6, LuxS*, *Pfs*) as listed in Table 1. The validity of each primer was confirmed by comparing its corresponding sequence with the database using BLAST (http://www.ncbi.nlm.nih.gov/BLAST/) [47]. Real-time PCR was performed as follows: initial denaturation step at 94 °C for 2 min, followed by 40 cycles of denaturation at 94 °C for15 s, primer annealing at 54 °C for 30 s and extension at 72 °C for 30 s. All samples were performed in triplicate for verification. Gene expression analysis was carried out using the 2-ΔΔCT method [48].

### Statistical analysis

Statistical analyses of biofilm formation were performed using two-way ANOVA and Tukey’s post-hoc tests. Gene expression data were analyzed by one-way ANOVA and Dunnett’s multiple comparisons test. Data were analyzed with Prism version 10 software (Graph Pad). All tests were performed with a confidence level of 95%.

## Results

### Planktonic inhibitory effects of oleuropein

The results of fluconazole sensitivity test for fungi showed that both *C. albicans* (MIC = 8 μg/ml) and *C. glabrata* (MIC = 64 μg/ml) were resistant to fluconazole. The results of microbroth dilution of *C. parapsilosis* ATCC 22019 (MIC = 2 μg/ml) and *C. krusei* ATCC 6258 (MIC = 16 μg/ml) confirmed the validity of this test.

The MIC result for oleuropein was 65 mg/ml for *C. albicans*, *C. glabrata* and *E. coli*, while the MFC/MBC was 130 mg/ml. The planktonic inhibitory effects of oleuropein (MIC, MFC/MBC) and growth inhibitory potential have been shown (Table 2).

**Table 2:** Inhibitory activity of oleuropein

Microorganisms	MIC (mg/ml)	MBC/MFC (mg/ml)
*Candida albicans*	65	130
*Candida glabrata*	65	130
*Escherichia coli*	65	130

### Antibiofilm activities of oleuropein

**Fig. 1:**
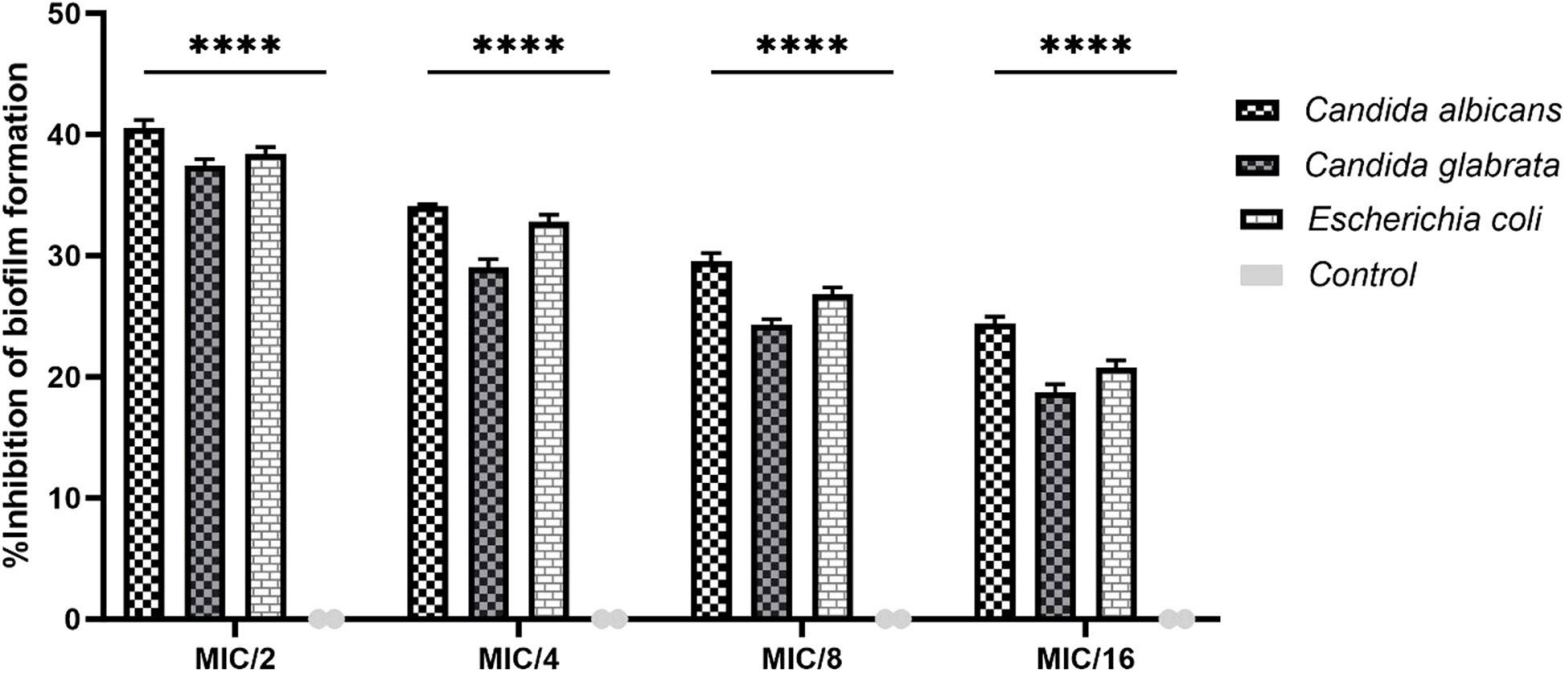
Percentage of inhibition of biofilm formation in *Candida albicans*, *Candida glabrata* and *Escherichia coli* after the treatment with different sub-MIC concentrations of oleuropein. Percent inhibition was determined relative to untreated control, which was considered as 0% inhibition. The error bars indicate SD. The asterisks represent statistical significance (****P < 0.0001)

**Fig. 2:**
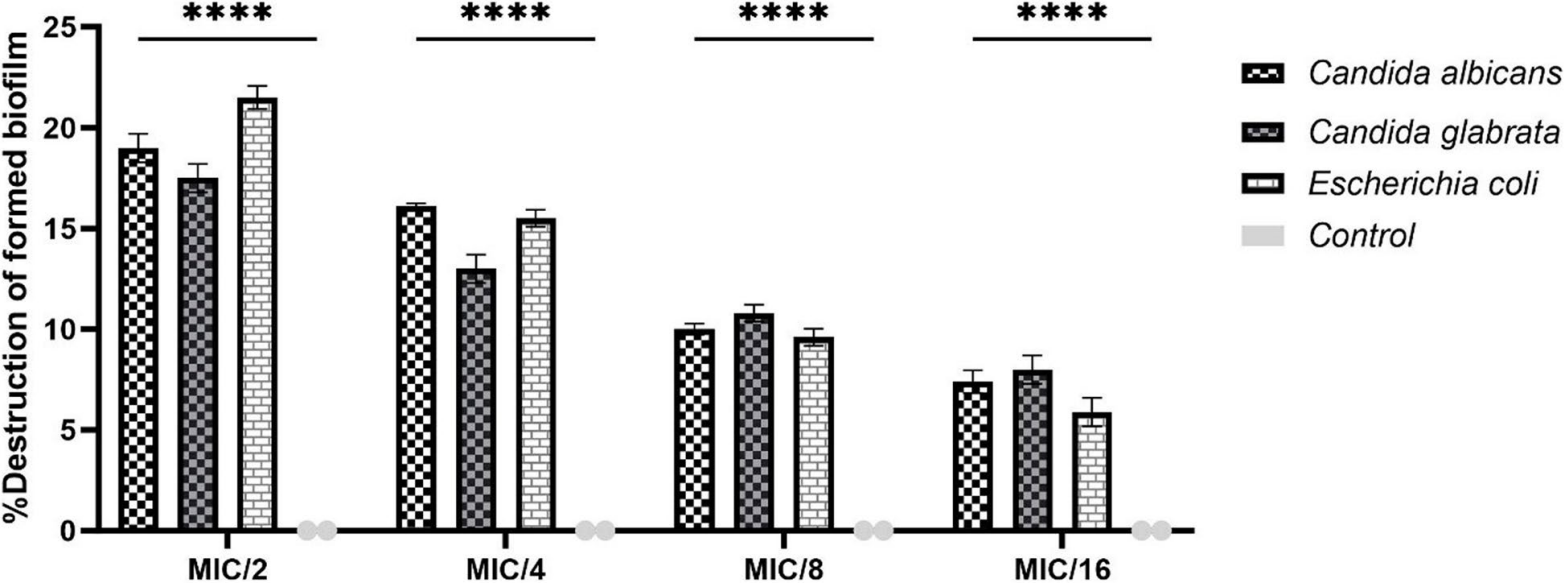
Percentage of destruction of formed biofilm in *Candida albicans*, *Candida glabrata* and *Escherichia coli* after the treatment with different sub-MIC concentrations of oleuropein. Percent destruction was determined relative to untreated control, which was considered as 0% destruction. The error bars indicate SD. The asterisks represent statistical significance (****P < 0.0001)

The antibiofilm activities of oleuropein were analyzed using the MTT assay as mean ± standard deviation (SD), and shown as the percentage of its inhibitory effect on biofilm formation (Fig. 1) and the percentage of destruction of the formed biofilm (Fig. 2). The mean ± SD of biofilm inhibition for *C. albicans* was 40.5 ± 0.5%, 34.1 ± 0.1%, 29.5 ± 0.5%, and 24.4 ± 0.4% at MIC/2, MIC/4, MIC/8, and MIC/16, respectively. For *C. glabrata*, the inhibition rate was 37.4 ± 0.4%, 29 ± 0.5%, 24.3 ± 0.3% and 18.7 ± 0.5% at the same concentrations. *E. coli* demonstrated inhibition rates of 38.4 ± 0.4%, 32.8 ± 0.4%, 26.8 ± 0.4% and 20.8 ± 0.4% at the same concentrations (Fig. 1). Also, *C. albicans* was destructed formed biofilm by 19 ± 0.5%, 16.1 ± 0.1%, 10 ± 0.2% and 7.4 ± 0.4%, while *C. glabrata* showed a reduction of 17.5 ± 0.5%, 13 ± 0.5%, 10.8 ± 0.3%, and 8 ± 0.5% in the concentrations of MIC/2, MIC/4, MIC/8, and MIC/16, respectively. For *E. coli*, the destruction rate was 21.5 ± 0.4%, 15.5 ± 0.3%, 9.6 ± 0.3% and 5.9 ± 0.5% in mentioned concentrations (Fig. 2).

The results show clear anti-biofilm effects in a dose-dependent manner. This dose-dependent relationship showed that higher doses of oleuropein are more effective in against biofilm formation and destroying formed biofilms. All four concentrations (MIC/2, MIC/4, MIC/8, and MIC/16) significantly inhibited biofilm formation and destruction of the formed biofilm by all three microorganisms studied (*p* < 0.0001).

### Structure of biofilm

**Fig. 3:**
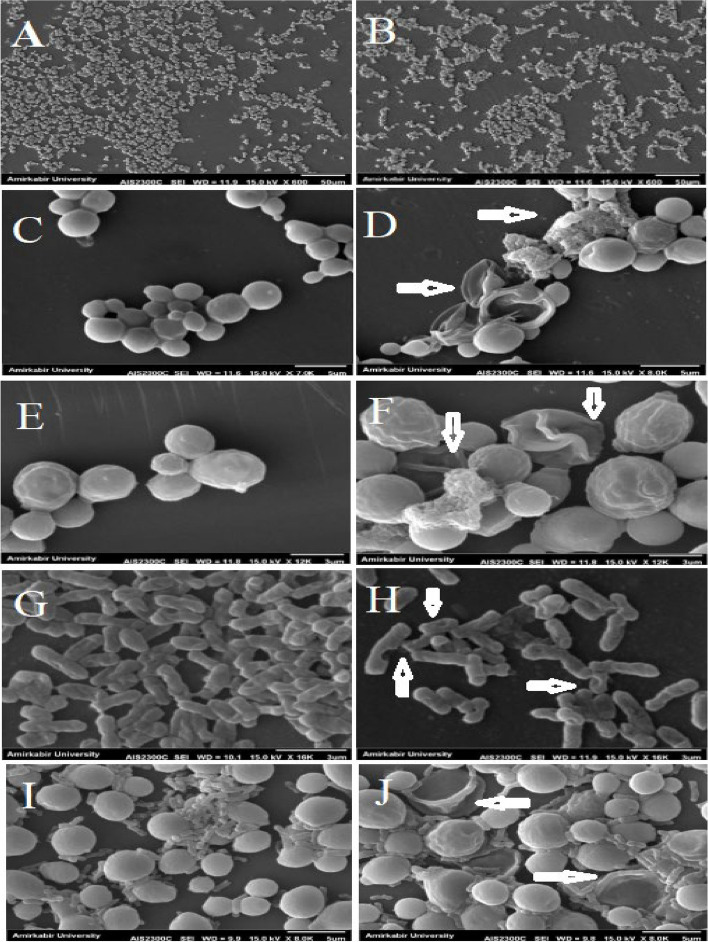
Scanning Electron Microscopy (SEM) images of different biofilms formed by *Candida* species and *Escherichia coli *treated/untreated by Oleuropein: (**A**, **C**) *C. albicans* without treatment; (**B**, **D**) *C. albicans* treated by MIC/2 of oleuropein, showing decrease in density of biofilm and disintegration of yeast (horizontal arrows); (**E**) *C. glabrata* without treatment; (**F**) *C. glabrata* treated by MIC/2 of oleuropein, showing increase in cell volume and destruction of yeast surfaces (vertical arrows); (**G**) *E. coli* without treatment; (**H**) *E. coli* treated by MIC/2 of oleuropein, showing decrease in cell aggregation and destruction of bacteria cell wall (arrows). (**I**) mixed biofilm of *C. albicans* and *E. coli* without treatment; (**J**) mixed biofilm of *C. albicans* and *E. coli* treated by MIC/2 of oleuropein showing destruction of yeast and *E. coli (arrows)*

By comparing the SEM photos of the biofilm cell treatment with the MIC/2 (32.5 mg/ml) concentration of oleuropein and the control (0 mg/ml), it could be seen that the outer cell membrane and cell morphology of the yeast and bacteria were deformed and the cells were more swollen. Additionally, the number of surface wrinkles on the yeast cells had increased. A decrease in cell accumulation was observed in all treated samples, especially in *E. coli*. Also, almost all the mentioned changes were observed in mix biofilm of *C. albicans* and *E. coli* (Fig. 3).

### Gene expression quantification of biofilm treated with oleuropein

**Fig. 4:**
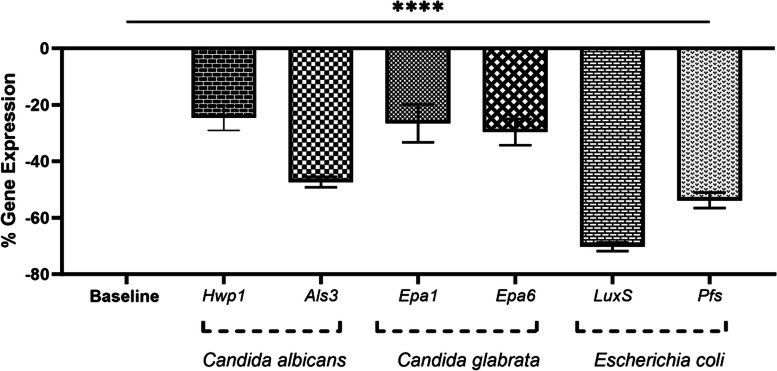
The percentage of gene expression related to biofilm formation in *Candida albicans* (*Hwp1, Als3*), *Candida glabrata* (*Epa1, Epa6*) and *Escherichia coli* (*LuxS, Pfs*) after treatment with MIC/2 (32.5 mg/ml) of oleuropein. The asterisks represent statistical significance (****P < 0.0001)

The effects of a MIC/2 concentration of oleuropein were measured on the expression of genes the affecting biofilm. This concentration of oleuropein caused a significant decrease in *Hwp1* (0.75-fold), *Als3* (0.52-fold), *Epa1* (0.73-fold), *Epa6* (0.70-fold), *LuxS* (0.29-fold) and *Pfs* (0.46-fold) (*p* < 0.0001) (Fig. 4). According to Fig. 4, the *luxS* gene showed the highest percentage of gene reduction among the tested genes, followed by the *pfs* gene from *E. coli* bacteria. On the contrary, the lowest percentage of gene reduction was observed in *Hwp1* gene of *C. albicans.* The value of percentage of down regulation in *Hwp1*, *Epa1* and *Epa6* was almost close to each other*.*

## Discussion

Medicinal plants have been used since ancient times to treat disease and their antimicrobial properties in various forms have been used as preservatives to maintain food quality, increase shelf life and disinfect the environment [49, 50]. Because of the increasing microbial resistance to common antifungal drugs, safer alternatives with fewer side effects should be considered for resistant drugs [51]. Oleuropein in virgin and extra virgin olive oil has high bioavailability. This compound has antioxidant, anti-inflammatory, anti-atherogenic, anti-cancer, antimicrobial and antiviral properties. It is commercially available as a food supplement in Mediterranean countries [52].

A previous study compared the inhibitory effect of oleuropein on several pathogenic microbes, including *C. albicans* (ATCC 10231) and *E. coli* (ATCC 8739). The highest required concentration of oleuropein was reported to be 0.6% (w/v). The use of this compound as a natural antimicrobial preservative in food, cosmetics, health and pharmaceutical products and for the treatment of infectious diseases such as superficial skin infections caused by pathogenic microorganisms has been shown to be very promising [53].

The current study investigated the antimicrobial and anti-biofilm activities of oleuropein against *C. albicans*, *C. glabrata* and *E. coli*. Due to the increasing use of fluconazole as treatment or prophylaxis for all types of illnesses, especially leukemia, bone marrow transplant recipients [54], neonates [55], and even patients with *Candida* vulvovaginitis [56], the prevalence of fluconazole-resistant *Candida* spp. is increasing worldwide [57]. Therefore, *C. albicans* and *C. glabrata* isolates used were those that showed high resistance to fluconazole. The results of the fluconazole sensitivity test for fungi showed that both *C. albicans* and *C. glabrata* were resistant to fluconazole.

Among the bacteria that pose the greatest threat to human health due to increased resistance to antibiotics are members of the *Enterobacteriaceae* family, especially *E. coli*. Among the different antibiotic resistance mechanisms of bacteria, the strongest and most diverse system is related to *E. coli* [58].

The MIC and MFC/MBC for oleuropein were found to be 65 mg/ml and 130 mg/ml, respectively. While most studies have focused on the antimicrobial effect of olive leaf extract, studies that have focused specifically on oleuropein are limited. In previous research, the inhibitory effect of oleuropein on *C. albicans* was demonstrated with an MIC value of 12.5 mg/ml and also showed that oleuropein targets the essential virulence factors of fungal infection [59].

In another study, when used alone, oleuropein exhibited mild antimicrobial activity against *Staphylococcus aureus* and *E. coli* planktonic cells with an MIC of 4.0 mg/ml [60]. Among microorganisms, *S. aureus* has been found to be the most sensitive to oleuropein extracts, while *E. coli* was the most resistant with MIC values ranging from 50 mg/ml to 12.5 mg/ml [61]. Oleuropein with concentration of 25 mg/ml has been found to inhibit the growth of *Listeria monocytogenes* (94%), *E. coli* (58%), and *Salmonella enteritidis* (36%) [62]. In other study, the MIC of olive leaf extract against *L. monocytogenes* was found to be 62.5 mg/ml, while the MIC of pure oleuropein was determined to be 25 mg/ml [63].

The results of these studies, along with the results of the current study, indicate that oleuropein has antimicrobial properties against different types of bacteria and fungi. It should be noted that the differences observed in the MIC results of this study and other studies can be explained due to the difference in the method of extraction and purification of oleuropein, as well as the difference in the amount of inherent or acquired resistance of the studied isolates.

SEM images showed a number of cells with wrinkled surfaces, perforated cells and collapsed cells. It could be concluded that oleuropein changed the fluidity of the cell membrane, which affected its permeability and that these surface changes affected adhesion of microbial cells to the target cells of the body. These observations were consistent with what has been previously reported in SEM images of *C. albicans* cells treated with olive leaf extract, which indicates became amorphous and deformations of the cell wall were manifested as inward invaginations [64]. It has been shown that oleuropein has a high affinity for sticking to the plasma membrane [65] and to negatively affect charged phospholipid membranes by influencing intermolecular interaction between oleuropein and anionic phospholipids in membranes at physiological pH and salt conditions. These interactions can result in changes to the cell membrane permeability, which some researchers attribute to the ortho-diphenol structure of oleuropein [66]. The results of SEM are consistent with reported mechanisms of action of oleuropein on surface permeability and of membrane destruction of microorganisms.

In the current study, the anti-biofilm activity of oleuropein against the common microorganisms of *C. albicans*, *C. glabrata* and *E. coli* was investigated. Biofilm formation by these microorganisms is very effective and is a factor in their pathogenesis [67]. Biofilm formation increases drug resistance against antibiotics by different mechanisms including delayed penetration of the antimicrobial agent into the extracellular matrix of the biofilm, reduced growth rate of organisms within the biofilm, or other physiological changes resulting from the interaction of organisms with a surface [68]; therefore, inhibiting or destroying biofilm is crucial. Figures 1 and 2 demonstrate that oleuropein significantly decreased biofilm formation by the investigated microbes at MIC/2, MIC/4, MIC/8 and MIC/16 concentrations (*p* < 0.0001). In Figs. 1 and 2, the highest percentage of biofilm inhibition or destruction was observed at MIC/2 concentration, and this value decreased with the reduction of oleuropein concentration in a dose-dependent. According to Fig. 1, *C. albicans* exhibited the most potent biofilm inhibitory effect in MIC/2 (40.5 ± 0.5%), MIC/4 (34.1 ± 0.1%), MIC/8 (29.5 ± 0.5%), and MIC/16 (24.4 ± 0.4%) compared to the two other microorganism isolates studied. *C. glabrata* showed the lowest percentage biofilm inhibitory effect in MIC/2 (37.4 ± 0.4%), MIC/4 (29 ± 0.5%), MIC/8 (24.3 ± 0.3%), and MIC/16 (18.7 ± 0.5%) compared to *E. coli* and *C. albicans*. In addition, in Fig. 2, the highest biofilm destruction at MIC/2 concentration was observed related to *E. coli* (21.5 ± 0.4%), while at MIC/4 concentration, it was associated with *C. albicans* (16.1 ± 0.1%). The highest biofilm destruction was showed at both MIC/8 and MIC/16 concentrations with *C. glabrata* (10.8 ± 0.3% and 8 ± 0.5%, respectively). On the contrary, *C. glabrata* exhibited the lowest percentage of biofilm destruction in MIC/2 (17.5 ± 0.5%), MIC/4 (13 ± 0.5%) and *E. coli* in MIC/8 (9.6 ± 0.3%), MIC/16 (5.9 ± 0.5%).

The antimicrobial properties of natural anti-biofilm agents have been utilized against biofilms [69]. For example, a study reported that concentrations of curcumin (0.1–2 mg/ml) effectively suppressed *Candida* Spp. Biofilms [70] or carvacrol suppressed *C. albicans* biofilm formation (80% at 2 mg/ml) [71]. Carvacrol inhibited *E. coli* biofilm formation at 125 μg/mL [41]. Hamzeh et al. demonstrated that the concentration of 1% thymol inhibits *E. coli* and *C. albicans* biofilms by 85.30 ± 0.52% and 84.40 ± 1.85%, respectively and it was also effective on polymicrobial biofilm [72].

Few studies have investigated the anti-biofilm properties of pure oleuropein against pathogenic microbes, but more studies have examined the anti-biofilm effect of olive leaf extract on different microorganisms and have demonstrated its effectiveness in reducing biofilm formation.

Oleuropein inhibited biofilm formation in *L. monocytogenes* at the concentration of 7.8 mg/ml. *Salmonella Enteritidis*, biofilm formation was inhibited by 74% at a concentration of 15.6 mg/ml [62].

The biofilm biomass of *S. aureus* (ATCC 25923) after treatment with oleuropein in the concentration range from MIC/16 to MIC (3 mg/mL) was significantly reduced by 24.40 to 91.95% compared to the control group [73].

Najee et al. demonstrated that the minimal biofilm eradication concentration *Olea europaea* fatty oil against *E. coli* ranged from 5.23 to 20.9 mg/ml and *C. albicans* from 2.61 to > 41.8 mg/ml [74]. Chetoui and Meski leaves extracts of olive cultures exhibited the most effective antibiofilm activity against *Pseudomonas aeruginosa*, *E. coli*, *Bacillus cereus*, *Enterococcus faecalis*, *S. aureus*, *S. aureus* MTR and *C. albicans*, with inhibition values of > 50% at MIC doses [75]. El-Sayed et al. demonstrated olive leaf extract at concentration 6.25 mg/ml had biofilm inhibitory effects by 82.2%, in multi-drug resistant *P. aeruginosa* and modulated QS genes [76]. 7.8 mg/ml of olive leaf extract were reduced both motility and biofilm formation of *L. monocytogenes* [63]*.* Other Studies have revealed the potential use of oleuropein as an additive to increase the antimicrobial effect of peracetic acid against biofilm formed by *S. aureus* and *L. monocytogenes* [55, 72]. The anti-biofilm results of oleuropein in all the above studies are consistent with the results of the present study. Only in single study, even though the effect of oleuropein was proven on *S. aureus* (ATCC 25923) and *E. coli* (ATCC 25922), no effect was observed on their biofilm formed on polystyrene microplates or stainless steel, which completely contradicts the results of the present study [60].

However, the effect of pure oleuropein on biofilm formation and destruction of the studied organisms by the present study has not previously been investigated. The current study showed that oleuropein significantly reduced the expression of genes related to biofilm formation in all three organisms (Fig. 4), leading to a disruption of the biofilm formation process. Theberge et al. [77] demonstrated the effect of a specific peptide on genes related to *C. albicans* biofilm (*Hwp1, NRG1, Eap1, EFG1*) using the RT-PCR method that resulted in a decrease in the growth of *C. albicans*. It was also demonstrated that the *Hwp1* gene encodes the hyphal cell wall proteins, an essential hyphal adhesion molecule vital for biofilm formation, which was shown to be expressed less in the presence of this peptide. Another study investigated the effect of probiotics on genes involved in *C. albicans* biofilm formation (*Hwp1, Als3*) and showed that these compounds significantly reduced the expression of several genes responsible for converting yeast into hyphae. The reduction rate of *Als3* (adhesion and virulence gene) was 70%, while *Hwp1* (hyphal wall protein crucial for biofilm formation) showed a reduction rate of over 99% [78]. Another study showed that the *Als3* gene was necessary for the formation of *C. albicans* biofilm [79]. It was found that the gene encodes a large cell surface glycoprotein with adhesive properties and that the protein expressed from it contributes to several essential functions in biofilm formation. The results of the study emphasized the importance of *Als3* in developing biofilm on silicon surfaces [79]. In Previous studies have not been investigated the effect of oleuropein on *Hwp1* and *Als3* genes in *C. albicans* biofilm. Liu et al. showed that exposure to 16 μg/mL of Eucarubostol E resulted in a significant decrease in the expression levels of *Hwp1* (5.89-fold) and *Als3* (9.09-fold) genes of *C. albicans* biofilms [80]. Shin et al. demonstrated that Zerumbone, a monocyclic sesquiterpene extracted from *Zingiber zerumbet*, at concentrations of 8, 16, and 32 μg/mL, downregulated expression levels of *Hwp1* by 2.57-, 3.1-, and 10.27-fold, respectively, and downregulated *Als3* gene by 1.67-, 3.36-, and 8.49-fold, respectively [81]. Haque et al. found that Sophorolipid (15 μg/mL), a glycolipid biosurfactant, downregulates the expression of hypha specific genes *Hwp1* (10-fold) and *Als3* (8.7-fold), which probably explains the inhibitory effect of this substance on the formation of hyphae and biofilm [82]. we observed that MIC/2 concentration (32.5 mg/ml) of oleuropein caused a downregulation in the expression of *Hwp1* (0.75-fold) and *Als3* (0.52-fold) in *C. albicans* biofilm. The reduction of *Hwp1* and *Als3* genes expression by oleuropein, as shown in previous studies, indicated the reduction of pathways related to biofilm formation, consequently leading to decrease in virulence in *C. albicans*.

Riera et al. [83] introduced the *Epa6* gene as a key gene for creating biofilm adhesion in *C. glabrata*. Very few studies have been conducted on the effect of herbal active substances on the *Epa1* and *Epa6* genes of *C. glabrata* biofilm. Nouri et al. showed 50 μg/mL concentration of Thymoquinone, extracted from of black cumin seed *Nigella sativa*, inhibited the biofilm formation of *C. glabrata* isolates, and 100% of the isolates downregulated expression of *EPA6* gene. This means that the biofilm’s ability was reduced for adhere to a specific structure [84]. In present study, a concentration of 32.5 mg/ml of oleuropein caused downregulation in *Epa1* (0.73-fold) and *Epa6* (0.70-fold) genes of *C. glabrata*.

Various studies have shown that phytochemicals disrupt the expression of QS-related genes including the *LuxS* and *Pfs* in *E. coli*. However, no study has been conducted on the impact of oleuropein on these genes. Lee et al. showed the decrease in the expression of *luxS* and *pfs* genes in response to treatment with 0.5% broccoli extract in *E. coli* [45]. Morgaan et al. showed that carvacrol downregulated the *luxS*, in 100% of the tested isolates with a fold reduction ranging from 1.06 to 1.62 [85].

Several articles have emphasized the effect of the *LuxS* and *Pfs* genes on the formation of *E. coli* biofilm and measured their effects on expression of different substances [26, 37, 41]. In this study, 32.5 mg/ml of oleuropein caused a significant decrease in the expression *LuxS* (0.29-fold) and *Pfs* (0.46-fold) (*p* < 0.0001). Sharifi et al. demonstrated that treating *E. coli* biofilm with 1.56 μg/ml of *Thymus daenensis* essential oil led to a decrease in the expression of *luxS* (6.13-fold) and *pfs* (4.12-fold). Similarly, treatment of the *E. coli* biofilm with 3.12 μg/ml of *Satureja hortensis* essential oil resulted in downregulation of *luxS* (5.11-fold) and *pfs* (3.98-fold) [37].

As observed in previous studies, the decreased expression of the analyzed target genes was associated with a decrease in biofilm formation. As a result, oleuropein played an important role in reducing the biofilm formation of the examined isolates by affecting the genes related to this process.

## Conclusion

The results of the current study both phenotypically and genetically showed that oleuropein has high anti-fungal and anti-bacterial properties. These properties can inhibit the formation of fungal and bacterial biofilms and even destroy them. Oleuropein was shown to have a significant effect on reducing the expression of the most important biofilm-forming genes. Of course, it should be noted that the concentration used in this study is higher than some previous researches. According to these results, oleuropein can be introduced as an antifungal and antibacterial herbal agent for topical and oral use and an environmental disinfectant. Of course, for therapeutic use, more studies are needed on possible side effects and other safety aspects of this combination. However, this substance might be significantly useful for inhibiting biofilms of medical stents, implants, endotracheal tubes, and catheters in to the future. It is suggested to study anti-biofilm activity of oleuropein against other clinically important *Candida* spp. such as *C. auris*.

## Data Availability

The data presented in this study are available from the corresponding author upon request.
